# Role of Matrix Metalloproteinase-2 in Eosinophil-Mediated Airway Remodeling

**DOI:** 10.3389/fimmu.2018.02163

**Published:** 2018-09-20

**Authors:** Yu Kuwabara, Tetsu Kobayashi, Corina N. D'Alessandro-Gabazza, Masaaki Toda, Taro Yasuma, Kota Nishihama, Atsuro Takeshita, Hajime Fujimoto, Mizuho Nagao, Takao Fujisawa, Esteban C. Gabazza

**Affiliations:** ^1^Allergy Center, Mie National Hospital, Tsu, Japan; ^2^Department of Pulmonary and Critical Care Medicine, Mie University Graduate School of Medicine, Tsu, Japan; ^3^Department of Immunology, Mie University Graduate School of Medicine, Tsu, Japan

**Keywords:** tissue remodeling, matrix metalloproteinases, bronchial asthma, eosinophils, airways, fibroblasts

## Abstract

Airway remodeling is responsible for the progressive decline of lung function in bronchial asthma. Matrix metalloproteinase-2 and fibroblast-to-myofibroblast transition are involved in tissue remodeling. Here we evaluated whether eosinophils play a role in fibroblasts-to-myofibroblasts transition and in the expression of matrix metalloproteinase-2. We co-cultured human eosinophils with human fetal lung fibroblast-1 cells, assessed the expression of remodeling-associated molecules by immunoassays and polymerase-chain reaction, and eosinophils-mediated migration of human fetal lung fibroblast-1 cells using a Boyden chamber. To clarify the participation of matrix metalloproteinase-2 in airway remodeling we administered bone marrow-derived eosinophils by intra-tracheal route to transgenic mice overexpressing the human matrix metalloproteinase-2. The expression of α-smooth muscle actin significantly increased in human fetal lung fibroblast-1 cells co-cultured with human eosinophils compared to controls. There was enhanced expression of matrix metalloproteinase-2 during fibroblast-to-myofibroblast transition. An inhibitor of matrix metalloproteinases blocked eosinophils-associated fibroblast-to-myofibroblast transition and increased migration of fibroblasts. The human matrix metalloproteinase-2 transgenic mice receiving adoptive transfer of mouse eosinophils exhibited increased inflammation and advanced airway remodeling compared to wild type mice. This study demonstrated that eosinophils induce fibroblast-to-myofibroblast transition, secretion of matrix metalloproteinase-2, accelerated migration of fibroblasts, and promote matrix metalloproteinase-2-related airway remodeling. These findings provide a novel mechanistic pathway for eosinophil-associated airway remodeling in bronchial asthma.

## Introduction

Bronchial asthma (BA) is a chronic inflammatory disease of the respiratory tract that may cause airway structural changes known as airway remodeling (1, 2). Therefore, bronchial asthma at early ages may increase the risk of developing chronic obstructive pulmonary disease in early adulthood (3). Airway remodeling is characterized by increased airway smooth muscle mass that may be the result of increased cell number (hyperplasia) and/or cell volume (hypertrophy), epithelial cell hyperplasia, goblet cell metaplasia, thickening of reticular basement membrane, and angiogenesis (4, 5). Among the multiple postulated mechanisms, epithelial-mesenchymal transition (EMT), and enhanced expression of transforming growth factor-(TGF)β1 are critical contributors to airway structural remodeling (6–8). Eosinophils are important sources of TGFβ1 in the asthmatic airway (9). We have previously demonstrated that eosinophils directly induce EMT of pulmonary epithelial cells by increasing the production of TGFβ1 (10). Some anti-asthmatic drugs such as montelukast and β2 adrenergic agonists suppress this eosinophil-induced EMT by attenuating the expression of TGFβ1 (11, 12). During EMT, epithelial cells differentiate into mesenchymal cells such as fibroblasts that are the predominant source of extracellular matrix proteins (13, 14). Fibroblast may also differentiate into myofibroblast, an intermediate phenotype between fibroblasts and α-smooth muscle actin (αSMA) cells that has greater ability to produce matrix components (13, 14). This fibroblast to myofibroblast transition (FMT) is also an important contributing factor in the pathogenesis of airway remodeling, TGFβ1 being the main inducer of FMT (15).

Matrix metalloproteinase (MMP)-2 is a member of a large family of calcium-dependent and zinc-containing endopeptidases that plays a critical function in the regulation of extracellular matrix degradation and deposition (16). Similar to other matrix metalloproteinases, besides modulating the structural components of the extracellular connective tissue, MMP-2 can also regulate cell division, differentiation, migration, apoptosis, and angiogenesis indirectly by activating proteins that affect cell function, or directly by binding to cell membrane-bound proteins linked to cell signal pathways (17). Several cells including fibroblasts, myofibroblasts, airway epithelial cells, and inflammatory cells can synthesize and secrete MMP-2 (17). During tissue injury, repair, and remodeling there is increased tissue concentration of matrix metalloproteinases including MMP-2 that facilitate the migration of α-actin smooth muscle cells and myofibroblasts and trafficking of inflammatory cells (17, 18). Direct interaction of bronchial epithelial cells from asthmatic subjects with migrating myofibroblasts fosters the ability of these mesenchymal cells to secrete extracellular matrix proteins including collagens (19). In addition, bronchial epithelial cells in contact with infiltrated eosinophils secrete high levels of TGFβ1 that induces EMT (10). However, the role of eosinophils in FMT and in MMP-2 secretion is unknown.

In the present investigation, we hypothesized that interaction with eosinophils promotes differentiation of fibroblasts to myofibroblasts and facilitates cell migration by increasing the expression of MMP-2 from epithelial cells.

## Materials and methods

### Reagent

L-glutamine, penicillin/streptomycin, Trizol Reagent, donkey anti-mouse IgG Alexa Fluor 488, and chicken anti-rabbit IgG-Alexa Fluor 594 were purchased from Invitrogen (Carlsbad, CA), Ham's F12K from Wako (Osaka, Japan) and fetal bovine serum (FBS) from Thermo Scientific. Anti-TGF-β1 monoclonal antibody (mAb) (1D11) from R&D Systems (Minneapolis, MN). Radioimmunoprecipitation (RIPA) lysis buffer was purchased from Santa Cruz Biotechnology (Santa Cruz, CA, USA). Horseradish peroxidase-conjugated goat anti-rabbit IgG, horseradish peroxidase conjugated goat anti-mouse IgG and Coomassie brilliant blue were from BIO-RAD (Hercules, CA, USA). Anti-CD16 and anti-CD14 bound micromagnetic beads, rabbit anti α-SMA were from Miltenyi Biotec (Auburn, CA), Sepasol-RNA I super G, gelatin, and casein from Nacalai Tesque (Kyoto, Japan) and doxycycline was from Sigma-Aldrich (St Louis, MO).

### Cell lines

Human fetal lung fibroblasts (HFL-1), provided by Riken (Kobe, Japan), were cultured in Ham's F12K supplemented with 15% (v/v) heat-inactivated FBS, 0.03% (w/v) L-glutamine, 100 IU/ml penicillin, and 100 μg/ml streptomycin. (penicillin–streptomycin, Gibco) in a humidified, 5% CO_2_ atmosphere at 37°C.

### Preparation of human eosinophils

After receiving informed consent, we isolated and purified human eosinophils from healthy volunteers by negative selection using anti-CD14 and anti-CD16 bound magnetic beads as previously described (20). The purity of eosinophils was more than 97% as measured by the Randolph's phloxine-methylene blue stain (20).

### Animals

Generation and characterization of the human MMP-2 transgenic (hMMP-2 TG) mouse were previously reported (21). We developed the hMMP2-TG mice originally using inbred C57BL/6 strain, and maintained by crossing homozygotes and/or heterozygotes. Wild-type mice from Japan SLC (Hamamatsu, Japan) that were maintained and bred in the same environment served as controls. We used 8–10 week-old female mice weighing 20–22 g. Mice were in a pathogen-free environment, kept on a constant 12:12-h light–dark cycle in a temperature- and humidity-controlled room and given water and standard mouse food *ad libitum*. The Mie University's Committee on Animal Investigation approved the experimental protocol (Approval No 25-20/Hen1-Sai; September 12, 2015).

### Co-culture experiment and morphological analysis

HFL-1 cells were cultured in 12-well plates until 60–70% cell confluence, then serum-starved overnight. Human eosinophils (1- or 2- or 4 × 10^5^ cells for 12-well plate) were added to the culture medium and incubated for 24 h or 48 h. Then, we removed the human eosinophils from adherent HFL-1 cells by gentle pipetting, and fixed the HFL-1 cells with 4% paraformaldehyde for 10 min at room temperature. After washing, HFL-1 cells were stained with mouse anti-MMP-2 and rabbit polyclonal anti α-SMA antibody followed by treatment with donkey anti-mouse IgG conjugated with AF488 and chicken anti-rabbit IgG conjugated with AF594 for 2 h at room temperature. 4, 6-Diamidino-2-phenylindole (DAPI) was used to stain nuclei for 5 min at room temperature. Fluorescent images were taken using a fluorescence microscope (I 71 Olympus) after appropriate cell washing.

To evaluate the role of TGFβ1 and the active form of MMP-2 during the interaction of eosinophils and fibroblasts, HFL-1 cells were cultured alone or co-cultured with eosinophils in the presence of anti-TGFβ1 monoclonal antibody (1D11; 10 μg /ml) or doxycycline (20 μg/ml), an inhibitor of matrix metalloproteinases.

### RT-PCR

After culture of HFL-1 cells alone or co-culture with eosinophils for 24 or 48 h and removal of eosinophils as described above, we extracted the total RNA from HFL-1 cells by the guanidine isothiocyanate procedure using Trizol Reagent. The RNA concentration and purity were determined by UV absorption at 260:280 using an Ultrospec 1100 pro UV/Vis spectrophotometer (Amersham Biosciences, NJ). RNA was reverse-transcribed (RT) using oligo-dT primers and then the DNA was amplified by polymerase chain reaction (PCR). The sequences of the primers are as follows: for human GAPDH, forward 5′-GTGAAGGTCGGACTCAAC GGA-3′ and reverse 5′-GGTGAAGACGCCAGTGGACTG-3′; for human α-SMA, forward 5′-GTGTTGCCCCTGAAGAGCAT-3′ and reverse 5′-GCT GGGACATTGAAAGTCTCA-3′: for human MMP-2, forward 5′-TACTGG ATCTACTCAGCCAGCAC-3′ and reverse 5′-CAGGATCCATTTTCTTCT TCACC-3′: for human TGF-β1, forward 5′-AAGACTATCGACATGGAG CTGG-3′and reverse 5′-GTATCGCCAGGAATTGTTGCTG-3′; for mouse GAPDH, forward 5′-TGGCCTTCCGTGTTCCTAC-3′ and reverse 5′-GAGTTG CTGTTGAAGTCGCA-3′; for mouse α-SMA, forward 5′-CAGGATGCAGAA GGAGATCAC-3′ and reverse 5′-TGTTGCTAGGCC AGGGCTAC-3′; for mouse collagen Iα1, forward 5′-TAAGGGTCCCCAATGGTGAGA-3′ and reverse 5′-GGGTCCCTCGACTCCTACAT-3′; for mouse periostin, forward 5′-CACGGC ATGGTTATTCCTTCA-3′ and reverse 5′- TCAGGACACGGTCAATGACAT-3′; for mouse MCP-1, forward 5′-ATGCAGGTCCCTGTCATGCTTC-3′ and reverse 5′-ACTAGTTCACTGTCACACTGGTC-3′. The condition of PCR was as follows: 26 to 35 cycles depending on the gene, denaturation at 94°C for 30 s, annealing at 65°C for 30 s, and elongation at 72°C for 1 min; at the end of these cycles, a further extension was carried out at 72°C for 5 min. We separated the PCR products on a 2% agarose gel containing 0.01% ethidium bromide and normalized the amount of mRNA against the GAPDH mRNA.

### Biochemical analysis

Commercial immunoassays kits were used to measure TGFβ1 and MMP-2 (R&D, McKinley Place, MN) following the manufacturer's instructions.

### Zymography

The proteolytic activity of MMP-2, MMP-7, MMP-9, and MMP12 was assessed by zymography using polyacrylamide gels and Coomassie brilliant blue staining as previously described (22). Gelatin (MMP-2, MMP-9) or casein (MMP-7, MMP-12) was used as substrate.

### Fibroblast migration

Migration of HFL-1 cells was assessed using a Boyden chamber (Corning, Kennebunk, ME) as previously described (23). The chemoattractant DMEM with 10% FBS was placed in the lower chamber, with an 8-μm-pore filter (Corning, Kennebunk, ME) coated with 0.1% gelatin or 0.1% casein on both sides (Becton Dickinson & Company Sparks, MD) between the upper and lower chambers. HFL-1 cells (1.0 × 10^6^ /ml in DMEM) and the appropriate number of eosinophils in the presence or absence of doxycycline (20 μg /ml) were loaded on the wells of the upper chamber followed by incubation for 24 h at 37°C in a 5% CO_2_ atmosphere. Then, cells adhered to the upper surface of the filter were removed by scraping and subsequently fixed and stained using Crystal Violet staining solution (Nacalai tesque, Kyoto, Japan). We counted the number of cells that migrated to the bottom surface of the filter in five randomly selected areas using a phase-contrast microscope.

### Adoptive transfer of mouse bone marrow-derived eosinophils into HMMP-2 TG and WT mice

To evaluate further the role of MMP-2 in airway remodeling, we transferred mouse bone marrow-derived eosinophils into hMMP-2 TG mouse and control WT mouse. Differentiated eosinophils from C57BL/6 mice bone marrow progenitors were prepared following a method previously reported (24). Briefly, we collected bone marrow cells from the femurs and tibiae, and lysed the red blood cells using a solution containing ammonium chloride, kalium hydrogen carbonate, and ethylenediaminetetraacetic acid. After washing, we cultured bone marrow cells in a medium containing RPMI 1640 with 10% FBS, 100 IU/ml penicillin and 10 μg/ml streptomycin, and 50 μM 2-mercaptoethanol (Sigma-Aldrich, St. Louis, MO) supplemented with 100 ng/ml stem cell factor and 100 ng/ml fms-like tyrosine kinase 3 (FLT3) ligand for 4 days. Thereafter, the medium containing stem cell factor and fms-like tyrosine kinase 3-ligand was replaced by medium containing 10 ng/ml recombinant mouse IL-5. After incubation with IL-5 for 3 days, we harvested the cells and confirmed differentiation to eosinophils by flow cytometry. The purity of eosinophils was more than 90 %. Under pentobarbital anesthesia, hMMP-2 TG and control WT mice received by intra-tracheal route either bone marrow-derived eosinophils (2 × 10^6^/cells in 100 μl saline) or sterile saline (100 μl) on days 0 and 7.

### Collection of samples

We collected samples after euthanasia of mice on day 9. BALF samples were drawn as previously described (10). The total number of cells in BALF was measured using a nucleocounter from ChemoMetec (Allerod, Denmark). The BALF supernatant was separated by centrifugation and stored at −80°C until use for biochemical analysis. For differential cell counting, BALF was centrifuged using a cytospin, the pelleted cells stained with May-Grunwald-Giemsa (Merck, Darmstadt, Germany) and the differential cell count was performed by an specialist. After completion of BALF collection, mice were thoracotomized and the pulmonary circulation flushed with saline followed by lung resection. The lung was perfused with 10% neutral buffered formalin, fixed in formalin for 24 h and then embedded in paraffin. Then, 5-μm-thick sections of lung specimens were prepared and stained with hematoxylin and eosin, Masson trichrome and periodic acid-Schiff (PAS) and examined under light microscopy (Olympus BX50 microscope, Tokyo, Japan). A blinded researcher randomly took the same number of photos of lung fields including bronchial walls from each mouse in all groups using an Olympus BX50 microscope or a fluorescence microscope (IX71 Olympus) combined with an Olympus DP70 digital camera (Tokyo, Japan). To assess the grade of peribronchial inflammation we counted the number of bronchus with and without infiltration of inflammatory cells including mononuclear and polymorphonuclear cells in lung tissue sections stained with hematoxylin & eosin. The grade of bronchial wall fibrosis was determined by measuring the areas that positively stained with Masson trichrome in relation to the total area of each lung field taken as 100% using the WinROOF image processing software (Mitani Corp., Fukui, Japan) for Windows. Hyperplasia of goblet cells was determined by measuring the areas that positively stained with PAS in relation to the total area of each lung field taken as 100%.

### Statistical analysis

Shown data are as the mean ± standard error of the means (S.E.M). The statistical difference between two variables was calculated by the Student's *t*-test, and between three or more variables by analysis of variance, using Fisher's protected least significant difference as *post-hoc* test. We performed the statistical analysis using Statview 5.0 software (Abacus systems, Berkley CA). A *p* < 0.05 was taken as statistically significant.

## Results

### Eosinophils stimulate TGFβ1 expression to induce FMT

**Figure 1:**
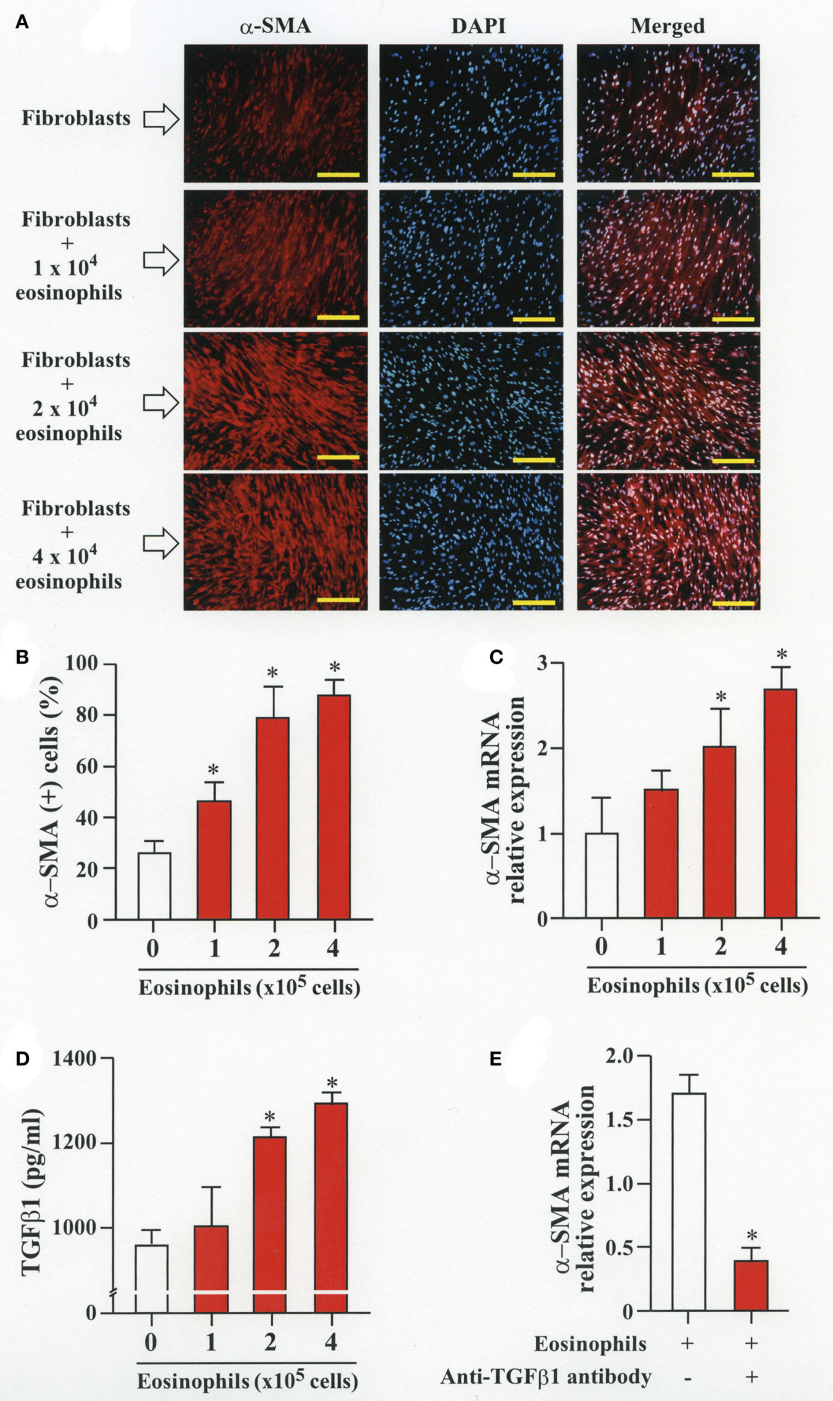
Human eosinophils induces fibroblast-to-myofibroblast transition (FMT). **(A)** Fibroblasts (HFL-1) co-cultured with eosinophils for 48 h. Immunofluorescence staining performed with anti-α-SMA (red) and diamidino-2-phenylindole (DAPI) for nuclei staining (blue) after washing out eosinophils. Scale bars indicate 200 μm. **(B)** Count of α-SMA positive cells. **(C)** Assessment of α-smooth muscle actin (α-SMA) mRNA relative expression in fibroblasts co-cultured with eosinophils by RT-PCR. **(D)** Protein level of TGFβ1 in the supernatant of HFL-1 cells co-cultured with eosinophils. **(E)** α-SMA mRNA expression in fibroblasts co-cultured with 2 × 10^5^ eosinophils in the presence or absence of anti-TGFβ1 neutralizing antibody by RT-PCR. Two independent experiments were performed and the results of one experiment are shown (*n* = 3 in each group). *P < 0.05 vs. absence of eosinophils.

To evaluate whether contact with eosinophils promotes transition of fibroblasts into myofibroblasts, we co-cultured fibroblasts (HFL-1) with human eosinophils for 48 h and evaluated the expression of α-SMA. The protein and the relative mRNA expression of α-SMA significantly increased in fibroblasts in the presence of eosinophils in a cell density-dependent manner (Figures 1A–C). The role of TGFβ1 in α-SMA expression was also evaluated. The concentration of TGFβ1 in the culture supernatant significantly increased in the presence of eosinophils in a cell density-dependent manner (Figure 1D). The addition of anti-TGFβ1 antibody to the co-culture significantly inhibited the eosinophil-associated increased expression of α-SMA (Figure 1E). These results suggest that co-culture with eosinophils promote TGFβ1 to increase α-SMA expression in fibroblasts.

### Eosinophils stimulate secretion of MMP-2 to activate latent TGFβ1 and increase α-SMA expression

**Figure 2:**
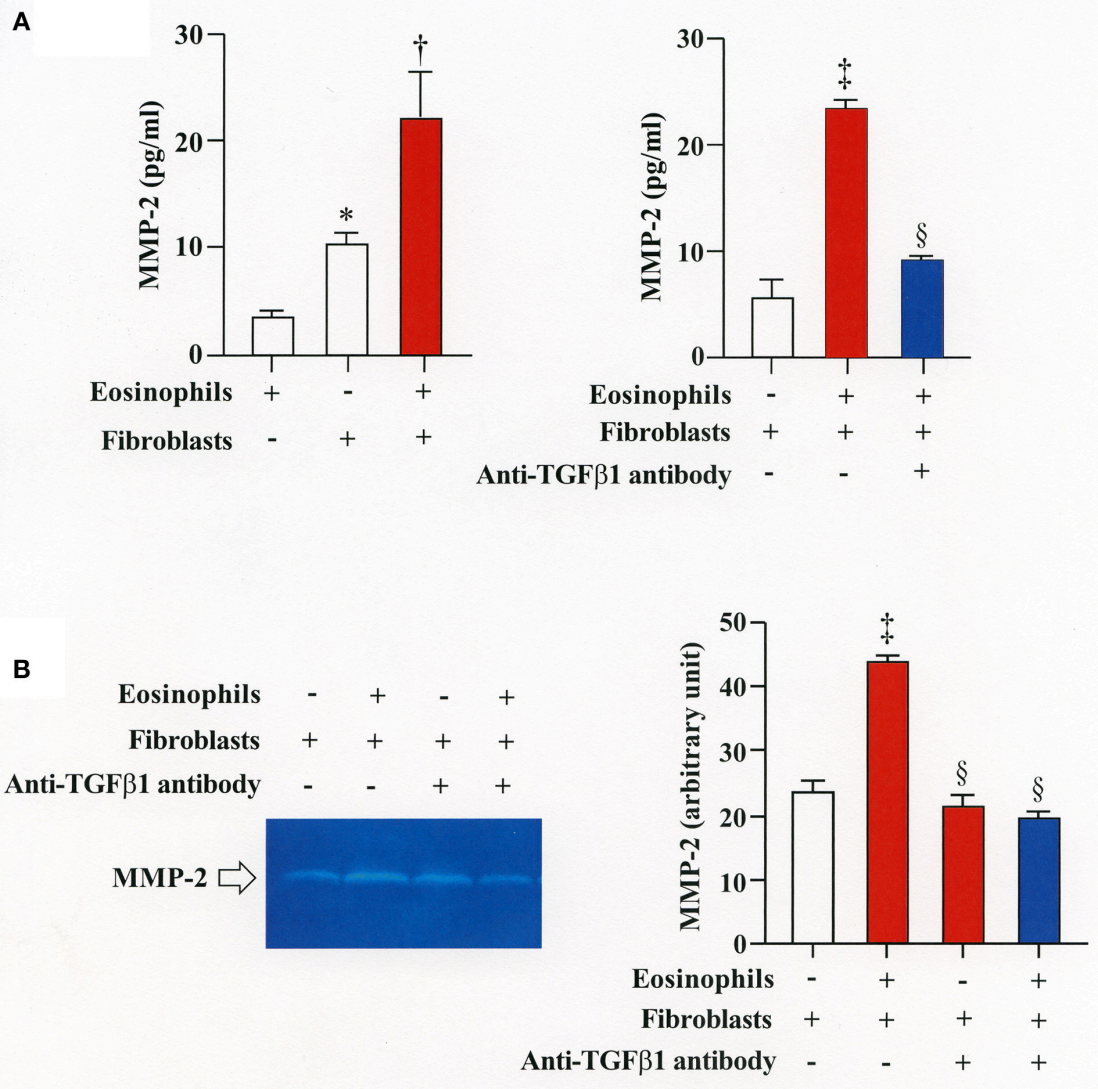
Stimulation of MMP-2 production by eosinophils is TGFβ1-dependent. **(A)** Assessment of MMP-2 level in cell supernatants from fibroblasts (HFL-1) co-cultured with eosinophils by enzyme immunoassays. **(B)** Assessment of MMP-2 level in cell supernatant from fibroblasts (HFL-1) co-cultured with eosinophils in the presence or absence of anti-TGFβ1 neutralizing antibody by gelatin zymography. The results of one experiment are shown (*n* = 3 in each group).*P < 0.05 vs. eosinophils (+)/fibroblasts (−); ^†^*P* < 0.05 vs. eosinophils (−)/fibroblasts (+);^‡^*P* < 0.05 vs. eosinophils (−)/fibroblasts (+)/anti-TGFβ1 (−); ^§^*P* < 0.05 vs. eosinophils (+)/fibroblasts (+)/anti-TGFβ1 (−).

**Figure 3:**
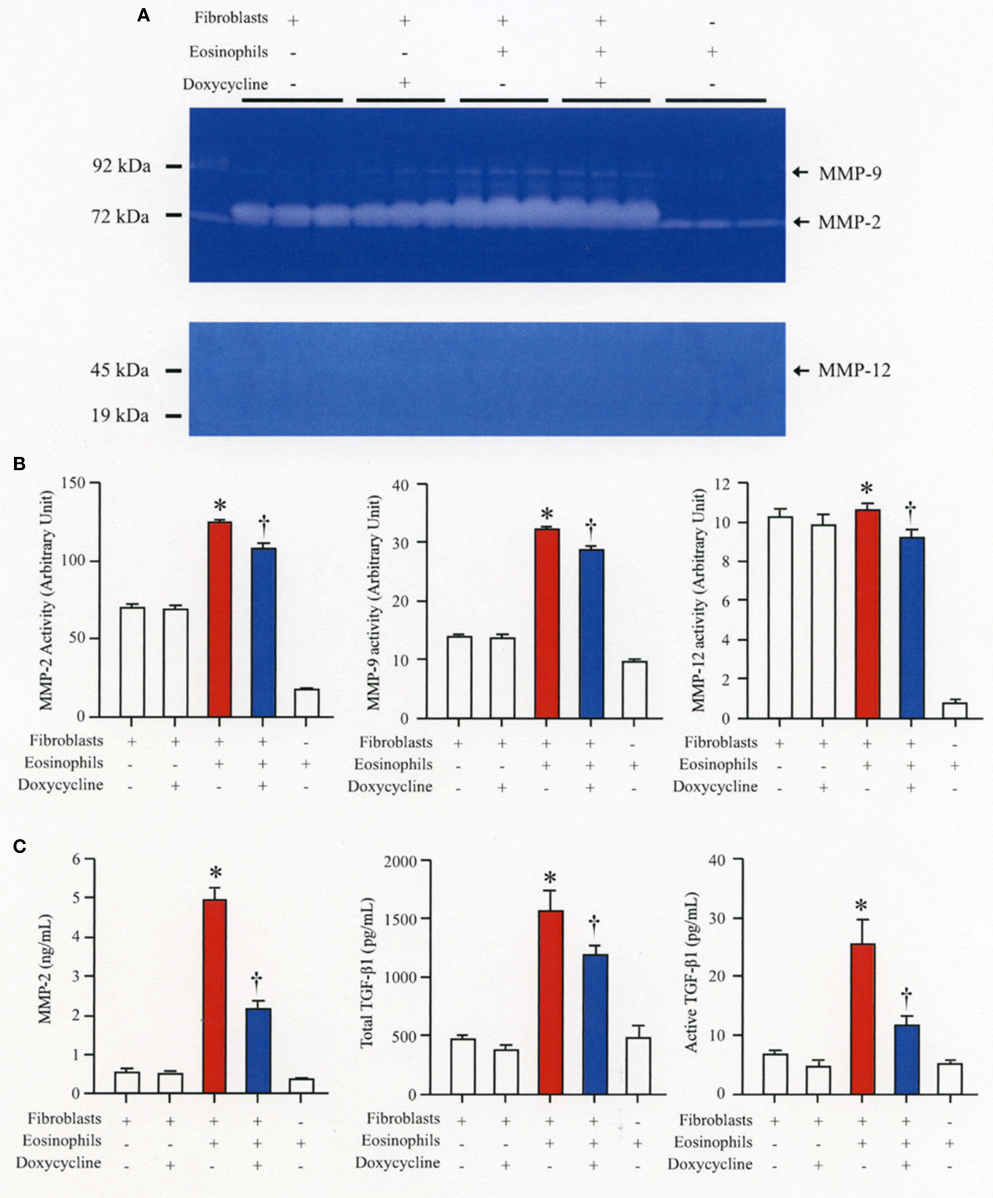
The broad-spectrum inhibitor of matrix metalloproteinase (MMP) doxycycline decreases activation of TGFβ1 during co-culture. HFL-1 cells were co-cultured with 2 × 10^5^ eosinophils in the presence or absence of 20 μg/mL of doxycycline for 48 h. **(A)** The activity levels of MMPs in the supernatant from HFL-1 cells co-cultured with eosinophils in the presence or absence of doxycycline were assessed by gelatin zymography (for MMP-2 and MMP-9) and by casein zymography (for MMP-7 and MMP-12). The bands corresponding to MMP-2, MMP-9, and MMP-12 are detectable but the band corresponding to the (19 kD) active form of MMP-7 is undetectable. **(B)** Quantification of MMP levels measured by using Image-J software. **(C)** MMP-2 and TGFβ1 (total and active form) levels were measured by commercial immunoassay kits. The results of one experiment are shown (*n* = 3 in each group). *P < 0.05 vs. fibroblast(+)/eosinophils(–)/doxycycline(–) group, fibroblast(+)/eosinophils(–)/doxycycline(+) group and fibroblast(–)/eosinophils(+)/doxycycline(–) group. ^†^*P* < 0.05 vs. fibroblast(+)/eosinophils(+)/doxycycline(–) group. ns, not significant.

**Figure 4:**
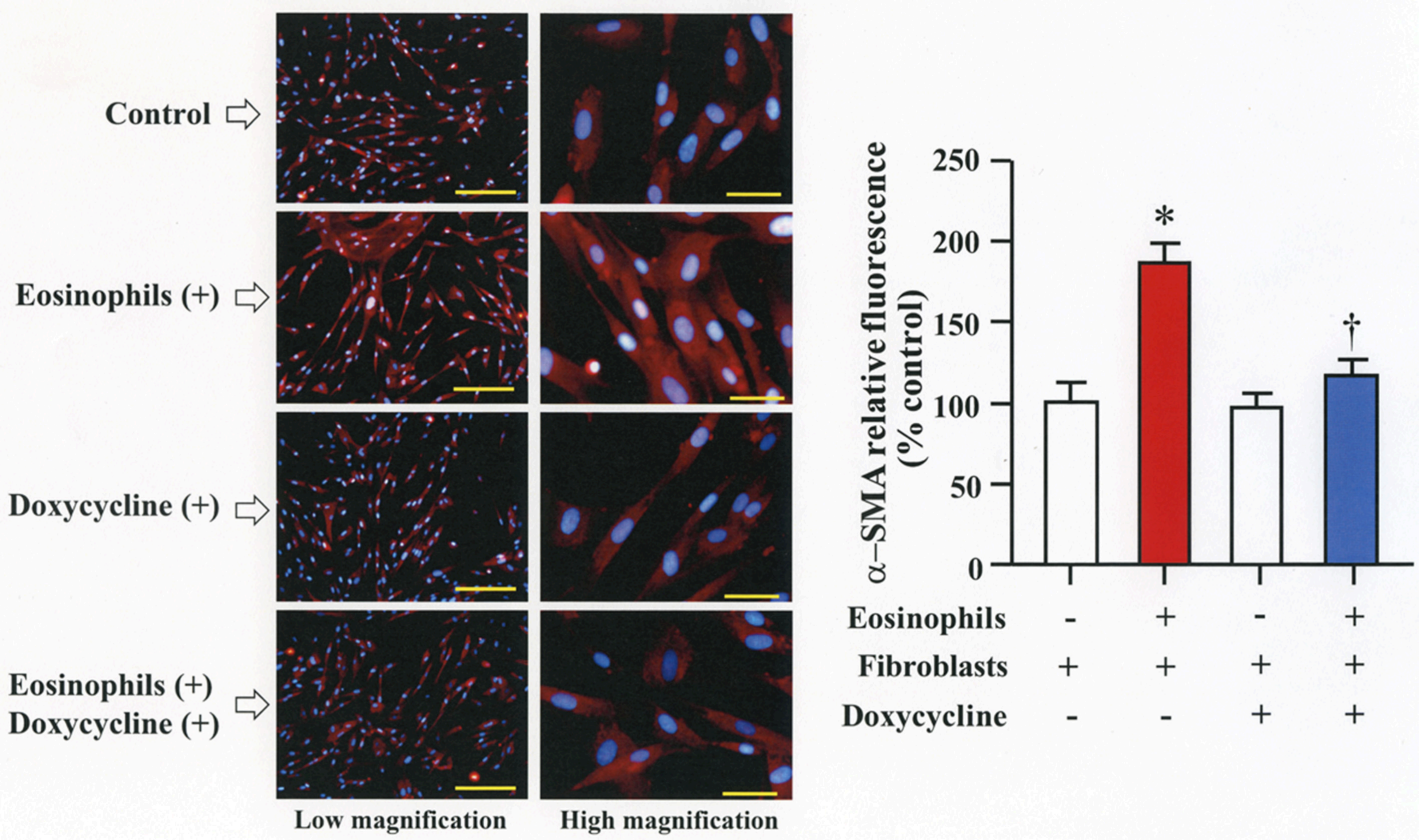
Doxycycline, a matrix metalloproteinase inhibitor, suppresses eosinophil-induced fibroblast-to-myofibroblast-transition. Fibroblasts (HFL-1) co-cultured with 2 x 10^5^ eosinophils in the presence or absence of 20 μg/mL of doxycycline for 48h. Immunofluorescence staining performed with anti-α-smooth muscle actin (SMA) (red color) and diamidino-2-phenylindole (DAPI) for nuclei staining (blue color) after washing out eosinophils. The scale bars indicate 200 μm (low magnification) and 50 μm (high magnification). Percentage of α-SMA positive assessed using Image-J software. Two independent experiments were performed and the results of one experiment are shown (*n* = 3 in each group). *P < 0.05 vs. eosinophils (–)/fibroblasts (+)/doxycycline (–); ^†^*P* < 0.05 vs. eosinophils (+)/fibroblasts (+)/doxycycline (–).

We then co-cultured fibroblasts (HFL-1) with human eosinophils for 24 h and evaluated the role of MMP-2 in TGFβ1 activation. Co-culture of fibroblasts with eosinophils significantly increased the total concentration of MMP-2 and the active form of MMP-2 in the culture supernatant but the increase was significantly suppressed in the presence of anti-TGFβ1 antibody, suggesting that TGFβ1 induces MMP-2 expression (Figures 2A,B). The proteolytic activity of MMP-7, MMP-9, and MMP-12 was also evaluated by zymography during co-culture. The activities of MMP-2, MMP-9, and MMP-12 were significantly increased in the co-culture supernatant compared to control and they were significantly suppressed in the presence of the matrix metalloproteinase inhibitor doxycycline (Figures 3A,B). The activity of MMP-7 was undetectable by zymography (Figure 3A). The concentrations of MMP-2, total and active TGFβ1 in the co-culture supernatant were also significantly suppressed in the presence of doxycycline compared to control (Figure 3C). Subsequently, to assess the importance of active MMP-2 in α-SMA expression, we co-cultured fibroblasts with eosinophils for 24 h in the presence or absence of doxycycline and assessed the expression of α-SMA by immunostaining with a specific antibody using an image software (Image-J software, National Institutes of Health, Bethesda, MD). We found significantly increased staining of α-SMA in cells co-cultured with eosinophils but a significant decreased staining in cells treated with doxycycline compared to controls, suggesting the need of active MMP-2 for the increased expression of α-SMA (Figure 4).

### Increased metalloproteinase activity during co-culture promotes migration of fibroblasts

**Figure 5:**
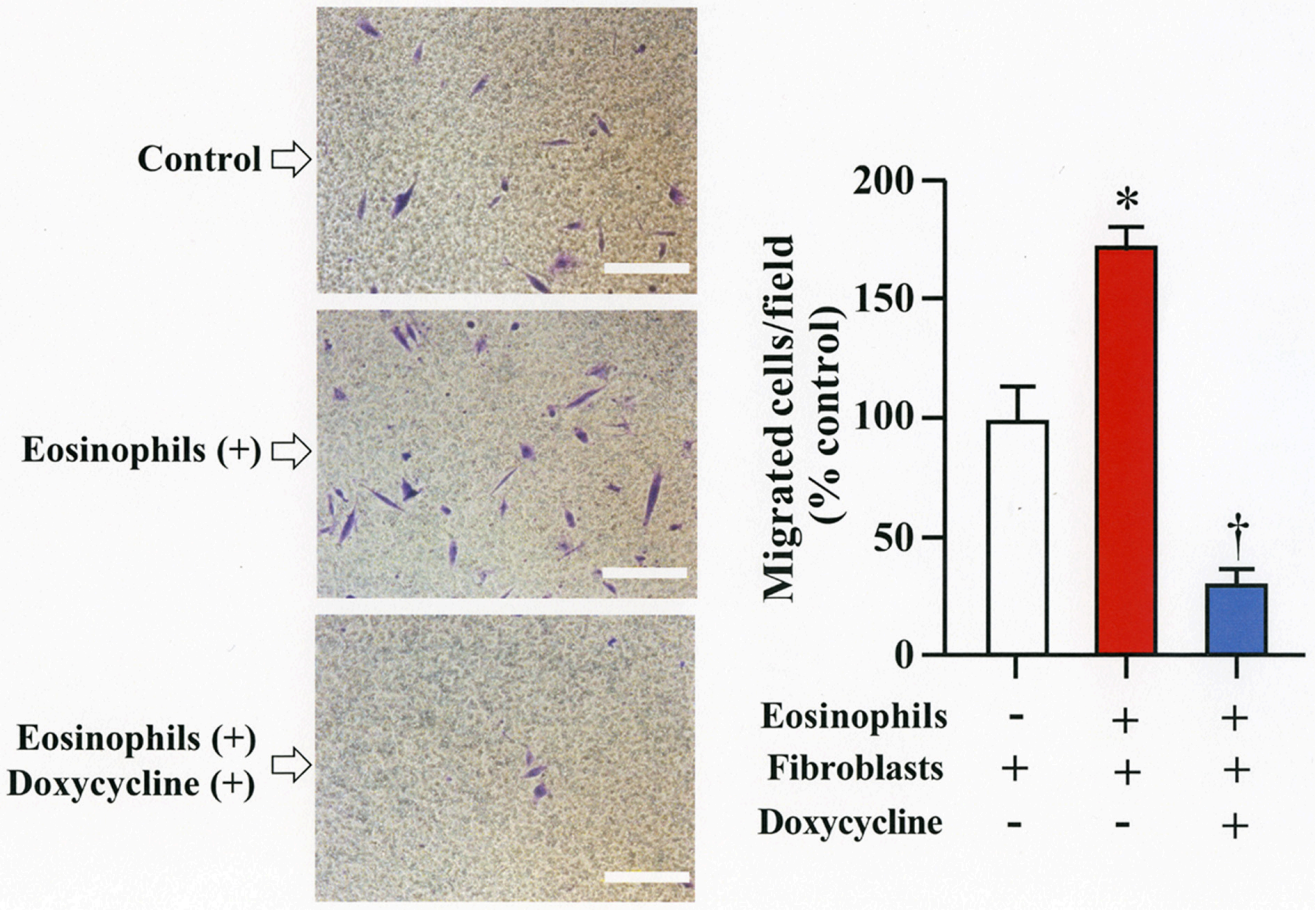
Doxycycline, a matrix metalloproteinase inhibitor, suppresses eosinophil-induced migration of lung fibroblasts. Migration of fibroblasts (HFL-1) evaluated by co-culturing with eosinophils in the presence or absence of 20 μg/mL of doxycycline for 24h using a Boyden chamber. Scale bars indicate 100 μm. Three independent experiments were performed and the results of one experiment are shown (*n* = 3 in each group). *P < 0.05 vs. eosinophils (–)/fibroblasts (+)/doxycycline (–); ^†^*P* < 0.05 vs. eosinophils (+)/fibroblasts (+)/doxycycline (–).

We assessed the number of fibroblasts that migrate in the presence of eosinophils using a Boyden chamber system. The number of fibroblasts that migrated through the chamber significantly increased in the presence of eosinophils compared to the number of cells that migrated in the absence of eosinophils. The addition of doxycycline significantly inhibited the migration of eosinophils compared to untreated cells, suggesting that increased level of matrix metalloproteinases during co-culture promotes migration of fibroblasts (Figure 5).

### Intra-tracheal administration of eosinophils promotes airway inflammation and remodeling in HMMP-2 overexpressing mice

**Figure 6:**
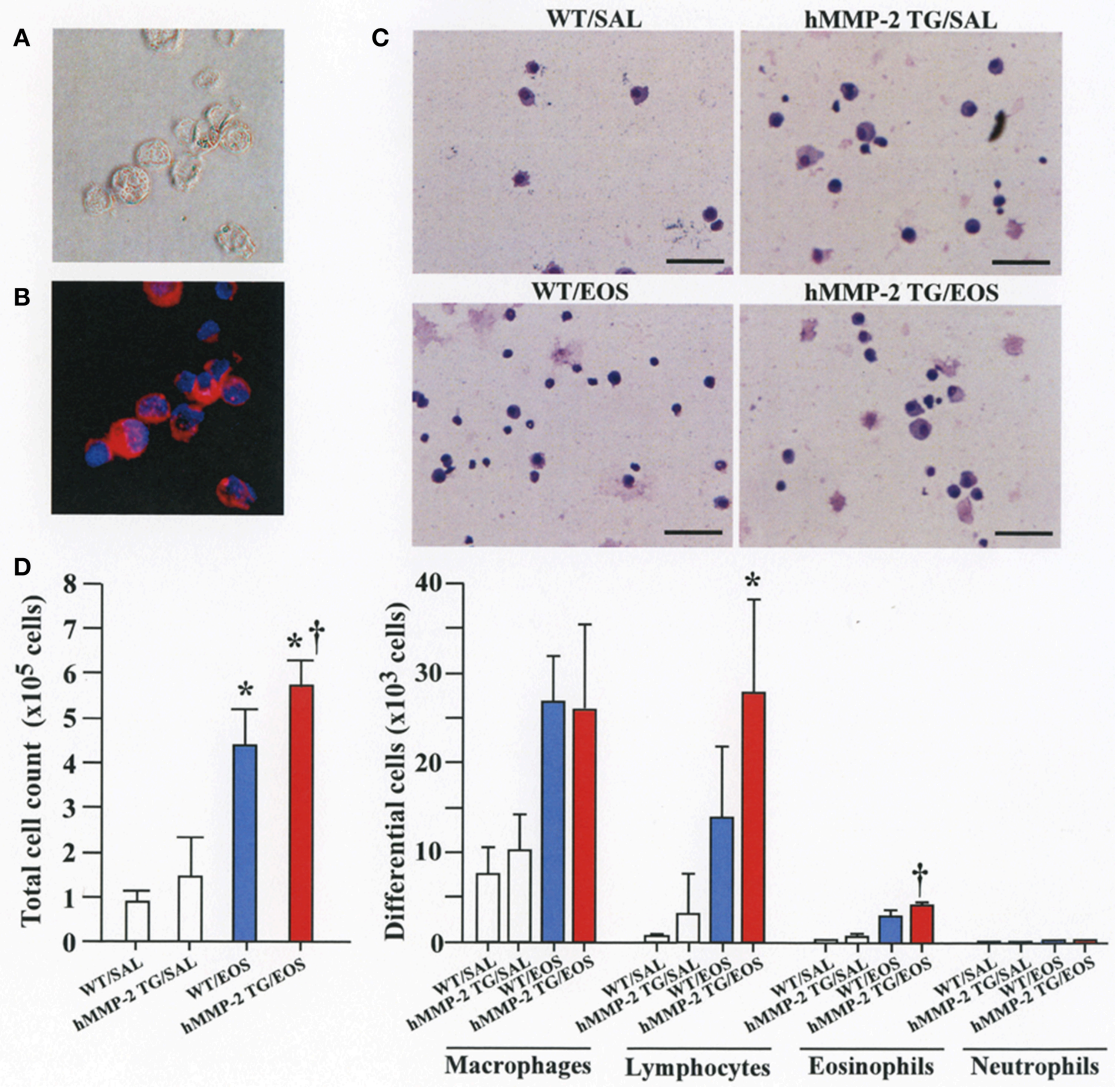
Inflammation in the airways walls of human MMP-2 transgenic mice after intra-tracheal administration of eosinophils. **(A,B)** Bone marrow-derived eosinophils prepared from wild type C57BL/6 mice. Phase-contrast micrograph **(A)** and eosinophils stained with EoProbe **(B)** eosinophil staining kit (magnification x400). Human MMP-2 transgenic mice and wild type mice received by intra-tracheal route bone marrow derived eosinophils. **(C)** The figure shows representative photos of bronchoalveolar lavage fluid (BALF) cells. **(D)** Total and differential cell count of BALF cells. Scale bars indicate 100 μm. *N* = 3 in each WT/SAL and hMMP-2/SAL group; *n* = 4 in each WT/EOS and hMMP-2 TG/EOS group. WT, wild type; SAL, saline; hMMP-2 TG, human matrix metalloproteinase transgenic; EOS, eosinophils. *P < 0.05 vs. WT/SAL or hMMP2 TG/SAL; ^†^*P* < 0.05 vs. WT/EOS.

**Figure 7:**
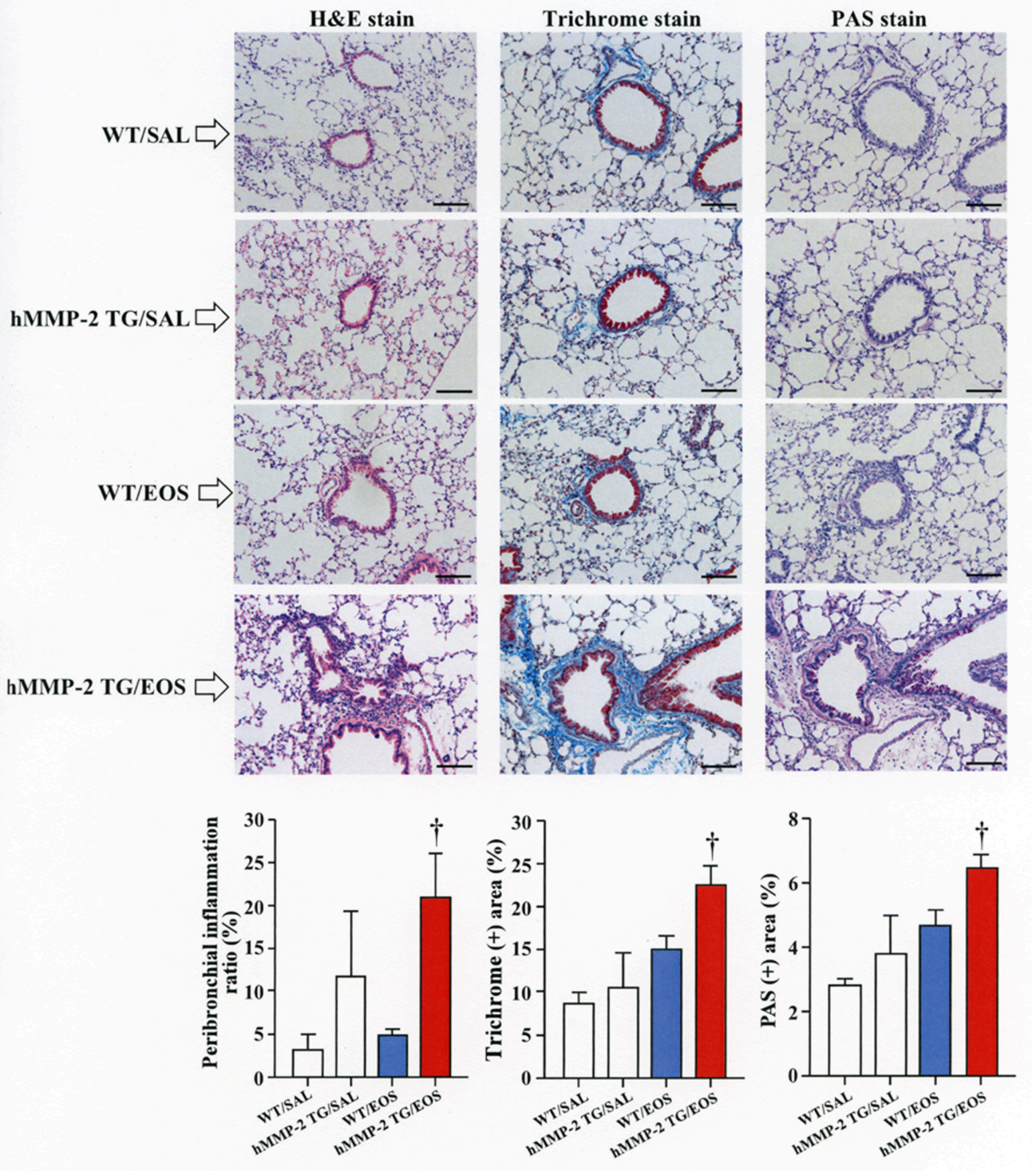
Lung fibrosis in human MMP-2 transgenic mice after intra-tracheal administration of eosinophils. Human MMP-2 transgenic mice and wild type mice received bone marrow derived eosinophils by intra-tracheal route. The figure shows representative photos of lung histological sections. The grade of peribronchial inflammation evaluated using a score system and the area of Masson trichrome and periodic acid–Schiff (PAS) stains using an image analyzer. Scale bars indicate 100 μm. *N* = 3 in each WT/SAL and hMMP-2/SAL group; *n* = 4 in each WT/EOS and hMMP-2 TG/EOS group. WT, wild type; SAL, saline; hMMP-2 TG, human matrix metalloproteinase transgenic; EOS, eosinophils. ^†^*P* < 0.05 vs. WT/EOS.

**Figure 8:**
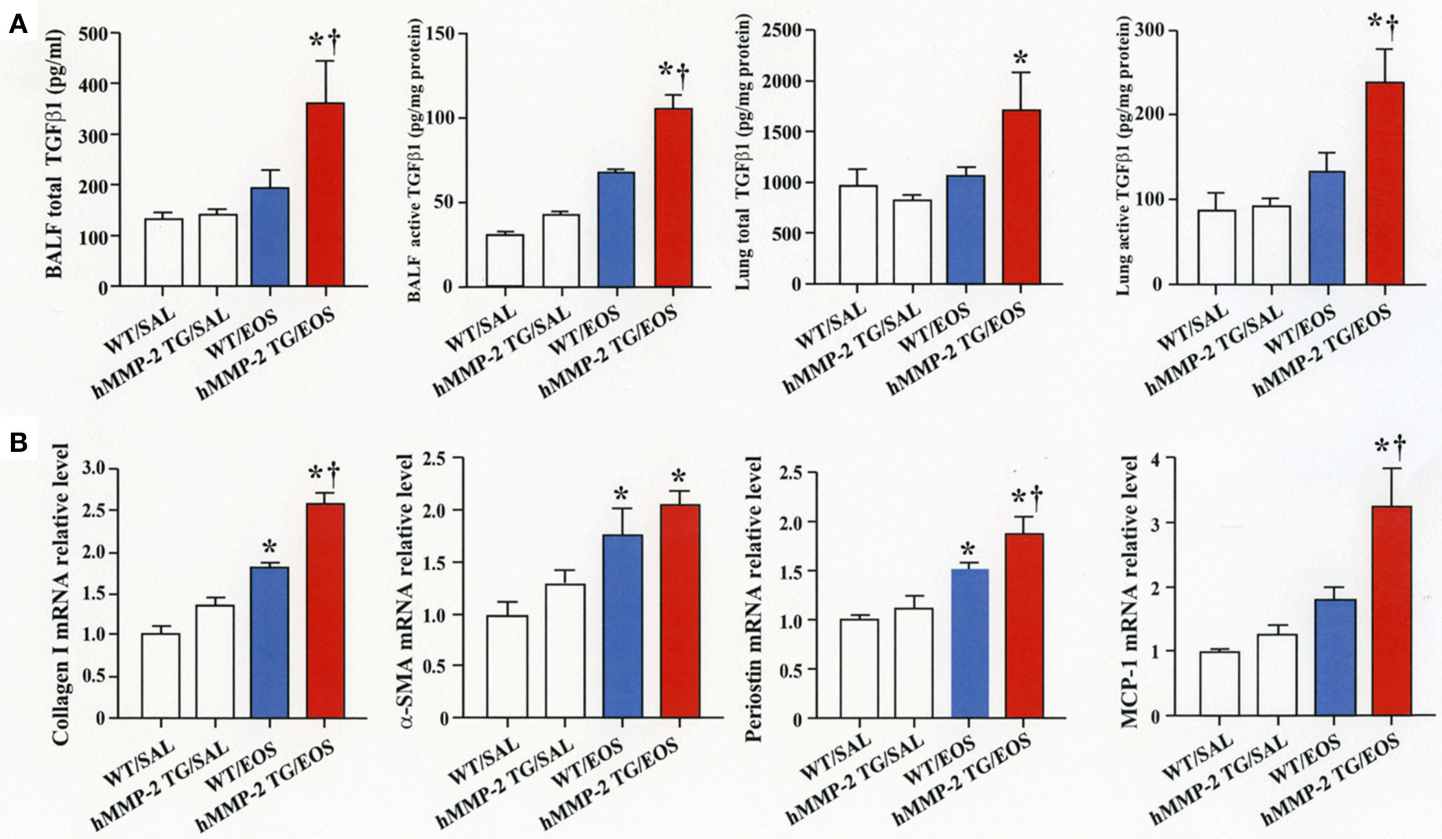
Expression of inflammatory and fibrotic markers in the lungs from human MMP-2 transgenic mice after intra-tracheal administration of eosinophils. Human MMP-2 transgenic mice and wild type mice received bone marrow derived eosinophils by intra-tracheal route. **(A)** TGFβ1 measured by enzyme immunoassays. **(B)** Parameters measured by RT-PCR. ^†^*P* < 0.05 vs. WT/EOS. *N* = 3 in each WT/SAL and hMMP-2/SAL group; *n* = 4 in each WT/EOS and hMMP-2 TG/EOS group. WT, wild type; SAL, saline; hMMP-2 TG, human matrix metalloproteinase transgenic; EOS, eosinophils. *P < 0.05 vs. WT/SAL or hMMP2 TG/SAL; ^†^*P* < 0.05 vs. WT/EOS.

To clarify the *in vivo* biological relevance of MMP-2 in the interaction between eosinophils and fibroblasts, we prepared mouse bone marrow-derived eosinophils and adoptively transferred to WT and hMMP-2 TG mice by intra-tracheal route (Figures 6A,B). WT mice transferred with eosinophils exhibited significantly enhanced cell counts with increased number of macrophages and lymphocytes in BALF compared to untreated WT mice (Figures 6C,D). The total cell count and the number of eosinophils significantly increased in BALF from hMMP-2 TG mice receiving intra-tracheal eosinophils compared to their WT counterpart group (Figures 6C,D). We also evaluated the number of bronchi with infiltration of inflammatory cells, the deposition of collagen and the area with mucin-secreting cells in all mouse groups. The results showed a significantly increased number of bronchial branches with cell infiltration, increased collagen deposition and enhanced areas with cells secreting mucin in hMMP-2 TG mice transferred with eosinophils compared to their WT counterpart (Figure 7). The BALF and lung tissue concentration of the total and active forms of TGFβ1 and the relative mRNA expression of collagen I, α-SMA, MCP-1, and periostin were significantly higher in hMMP-2 TG/EOS mice than in WT/EOS mice (Figures 8A,B). These results suggest that MMP-2 is an important mediator of inflammation and tissue remodeling induced by eosinophils in the airways.

## Discussion

### Eosinophils in FMT

**Figure 9:**
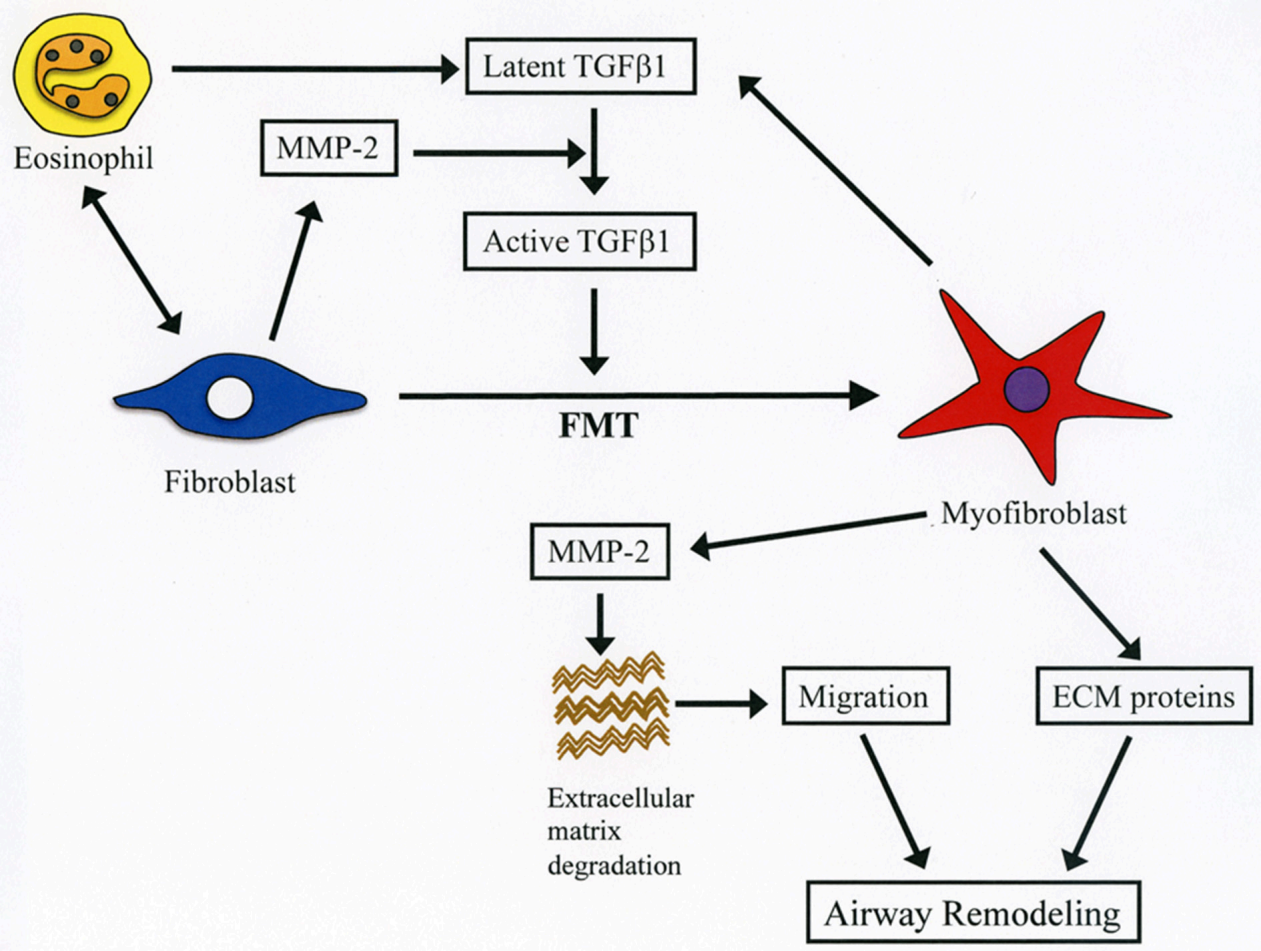
Summary of the role of MMP-2 in eosinophil-mediated airway remodeling based on current study findings. During interaction of eosinophils with lung fibroblasts, they are activated releasing latent form of TGFβ1. Activation of fibroblasts after contacting with eosinophils leads to increased production of MMP-2, which cleaves the latent form of TGFβ1 into its active form. Activated TGFβ1 induces fibroblast-to-myofibroblast transition (FMT), and myofibroblasts secrete both TGFβ1 and MMP-2 further enhancing FMT. The increased level of MMP-2 breakdown extracellular matrix proteins leading to enhanced migration of myofibroblasts into the airways. Infiltrated myofibroblasts synthesize and produce new extracellular proteins (e.g., collagen, fibronectin) inducing airway remodeling.

The cellular composition of chronically inflamed airways in allergic BA is characterized by the presence of high number of inflammatory cells including CD4^+^ T cells, eosinophils, basophils and mast cells (25). Following activation CD4^+^ T cells release inflammatory mediators and Th2 cytokines such as IL-5 that can activate and further stimulate recruitment of eosinophils into the lungs (25). The persistent airway inflammation ends up causing unduly tissue remodeling that has well-recognized clinical consequences (26). Eosinophils may directly participate in the process of airway remodeling by releasing pro-fibrotic factors especially TGFβ1, or indirectly by stimulating the secretion of TGFβ1 from bronchial epithelial cells through direct contact (10, 12, 27). TGFβ1 promotes fibrosis of bronchial walls by enhancing the synthesis and secretion of matrix proteins (collagens, proteoglycans, tenascins, periostin), by stimulating the secretion of profibrotic factors (e.g., platelet-derived growth factor) and chemotaxis of fibroblasts and by promoting EMT and FMT (25, 28). The net result of these effects is the local accumulation of myofibroblast, which is the primary source of type I and II collagen in fibrotic diseases (29). The contractile property of myofibroblasts enables them to participate in the contraction of the asthmatic airway (30). In the present study, we found that direct interaction with eosinophils increases the differentiation of fibroblasts to myofibroblasts as demonstrated by the increased expression of α-SMA in lung fibroblasts after co-culture with eosinophils. Upregulated expression of TGFβ1 appears to induce FMT in our experimental model as we found a dramatic increase of TGFβ1 expression in the co-culture supernatant. In most cells TGFβ1 is secreted in latent form and it is subsequently activated by various proteases including MMP-2 (31). The concomitant increase of MMP-2 in the co-culture supernatant points this gelatinase as the most probable activator of TGFβ1 in our culture system. Inhibition of α-SMA expression by doxycycline suggests the requirement of MMP-2-mediated activation of TGFβ1 for the induction of FMT (Figure 9). Regarding the cell source of mediators, based on the results described in Figures 2, 3, although eosinophils and fibroblast can equally contribute to TGFβ1 secretion, fibroblasts appear to be more potent secretors of MMP-2 than eosinophils. In brief, increased FMT induced by TGFβ1 released during interaction of eosinophils with fibroblasts is a new mechanism by which eosinophils can exert a deleterious role in airway remodeling.

### MMP-2 in cell migration

Cell migration is an important process during inflammation and wound healing (32). Cells need to overcome several barriers before reaching sites of tissue injury and wound repair (32). Tissue -trafficking of cells involves an exquisite interplay of different factors including cytokines, growth factors, matrix components, matrix metalloproteinases, chemokines, adhesion molecules, and cell receptors (29, 32). Of these, the role of matrix metalloproteinases such as MMP-2 are essential because they degrade extracellular connective tissue components and activate chemokines and growth factors by limited proteolysis and thus facilitating the motility and passage of leukocytes and fibroblasts (Figure 9) (33). There is an excessive accumulation of eosinophils and fibroblasts in the remodeled airways of asthmatic patients but whether interaction of both cells further enhances the population of fibroblasts in the lung is unknown (25, 34). In the present study, we found that co-culture with eosinophils increases the migration of fibroblasts and that doxycycline blocks this process implicating a role for MMP-2 in the mechanism. It is worth noting that the inhibitory activity of doxycycline on MMPs was less pronounced in the zymography study compared to its inhibitory activity in the α-SMA production and fibroblast migration assays, suggesting that doxycycline regulates other proteins or pathways in addition to MMPs. To assess *in vivo* the biological relevance of eosinophils and MMP-2 participation in airway remodeling we adoptively transferred murine eosinophils into the airways of mice overexpressing the human MMP-2. We found that hMMP-2 TG mice receiving intra-tracheal eosinophils have significant lung structural changes characterized by peribronchial inflammation, fibrosis, and hyperplasia of mucin-producing cells in association with enhanced lung expression of TGFβ1, MCP-1, periostin, α-SMA and collagen compared to WT mice treated in a similar manner (Figure 9). These observations suggest that interaction of eosinophils with fibroblasts during eosinophilic inflammation can further contribute to airway remodeling by promoting MMP-2-mediated migration of fibroblasts. Interestingly, in the *in vivo* experiment, there was a significantly increased number of eosinophils in hMMP-2 TG mice treated with intra-tracheal eosinophils compared to their WT counterparts. MMP-2 can prolong cell survival by inhibiting apoptosis (21); therefore, it is conceivable that prolongation of eosinophil survival in an environment with high MMP-2 level explains the difference in the BALF number of eosinophils between MMP-2 TG and WT mice.

## Conclusion

The present study shows for the first time that interaction of eosinophils with fibroblasts promotes the differentiation of fibroblasts to myofibroblasts by stimulating the expression of TGF-β1 and the migration of fibroblasts by increasing the expression of MMP-2 (Figure 8). These novel findings may explain the detrimental role of eosinophils in airway remodeling.

## Author contributions

CD-G and KN performed and prepared the mouse model. HF and TY evaluated the lung pathological findings. MT, YK, KN, and AT measured and analyzed several parameters. YK, HF, and MT performed the *in vitro* cell study. TK, EG, MN, and TF conceived and designed the experiments, analyzed the data, and contributed with critical revision and interpretation.

### Conflict of interest statement

YK received a grant from GlaxoSmithKline Japan and CD-G received a grant from the Ministry of Education of Japan (Kakenhi No. 17K08442). EG and TK have a patent on the hMMP-2 TG mouse used in this study. The remaining authors declare that the research was conducted in the absence of any commercial or financial relationships that could be construed as a potential conflict of interest.
